# First case of *Brucella suis* biovar 1 infection in a dog in Switzerland

**DOI:** 10.1007/s11259-026-11491-y

**Published:** 2026-09-04

**Authors:** Ezgi Akdesir, Michèle Spörri, Natascha Gross, Philipp Schneider, Isabelle Brodard, Simon Feyer, Gudrun Overesch, Sonja Kittl

**Affiliations:** 1 Institute of Veterinary Bacteriology, Vetsuisse Faculty, University of Bern, Bern, Switzerland; 2 Bessy’s Small Animal Clinic, ZN IVC Evidensia Schweiz AG, Watt/Regensdorf, Switzerland

**Keywords:** Canine brucellosis, Diagnostics, Lipopolysaccharide, Serology, Whole genome sequencing

## Abstract

As one of the most common zoonoses globally, brucellosis threatens not only animals but also humans with *Brucella (B.) melitensis*,* B. abortus* and *B. suis* biovar 1&3, being more virulent for humans than *B. canis*, *B. ovis* and *B. suis* biovar 2. Canine brucellosis is caused mainly by *B. canis*, however, *B. suis* infections in dogs have been reported sporadically in Europe and more frequently in Australia. *B. suis* infection in dogs is mainly associated with hunting or wild-animal exposure and rarely with consumption of commercial raw meat products. In Switzerland, *B. canis* is sporadically diagnosed in dogs and *B. suis* biovar 2 is present in wild boar and brown hare. We report a case of brucellosis in a dog from Switzerland, neither having a history of hunting nor travel-associated risk of exposure. The intact male dog showed clinical signs (fever and epididymitis) consistent with brucellosis, which was confirmed by culture and molecular methods in urine and by serological methods. Culture and subsequent whole genome sequencing revealed the isolate as *B. suis* biovar 1, ST-14. The most closely related strain was shown to be a strain isolated in 2021 from a dog in Germany. Based on the lack of any previous report, infection by *B. suis* in dogs has not previously been diagnosed in Switzerland. This case highlights the need for vigilance regarding this less expected pathogen of high zoonotic importance in dogs.

## Background

*Brucella* (*B.*) species are facultative intracellular, fastidious bacteria, causing a plethora of clinical signs in animals including abortion, orchitis and osteoarticular disease. One of the most significant virulence factors of *Brucella* spp. is lipopolysaccharide (LPS), an essential structural component of Gram-negative bacterial cell walls. *Brucella* species produce two types of LPS: smooth forms (sLPS), which contain complete LPS in the outer membrane (comprising lipid A, a polysaccharide core, and an O-polysaccharide), and rough phenotypes (rLPS), which lack the polysaccharide O-chain. Classical smooth *Brucella* species include *B. abortus*,* B. melitensis*, and *B. suis*, while *B. canis* and *B. ovis* naturally exhibit rough phenotypes (Stranahan and Arenas-Gamboa 2021).

Canine brucellosis is caused mainly by *B. canis* and less commonly by other *Brucella* species like *B. suis* (Woldemeskel 2013; van Dijk et al. 2018; Kneipp et al. 2021, 2023a, b, 2025; Aurich et al. 2023; Girault et al. 2023; Dadar et al. 2025). *B. suis* biovar 1 has more zoonotic potential than the *B. suis* biovar 2, which is the most common type of *B. suis* circulating in wildlife in most European countries (Godfroid et al. 2005). Switzerland is free from brucellosis in farm animals, however, *B. suis* biovar 2 is present in wild boar (*Sus scrofa*) and brown hare (*Lepus europaeus*) as in most European countries (Wu et al. 2012; Kreizinger et al. 2014; Ferreira et al. 2017; Macías Luaces et al. 2023; Ruano et al. 2025). *B. suis* biovar 1 has never been reported in wild animals in Switzerland. There are also sporadic cases of *B. canis* infections mainly in imported dogs, but true numbers and the burden of the disease in Switzerland, as in Europe, remains unknown (Buhmann et al. 2019), as the disease is not reportable in most countries and the dog population is not screened systematically via harmonized monitoring programs. However, awareness about canine brucellosis is increasing and importance of control measures is being emphasized (Buhmann et al. 2019; Boyden 2022; De Massis et al. 2022). For instance, authorities in United Kingdom started pre-import mandatory testing for *B. canis* in dogs from high-risk countries (British Veterinary Association 2025).

The clinical presentation of canine brucellosis is highly variable, often manifesting as reproductive disorders (abortion, epididymitis, orchitis), osteoarticular disease (septic arthritis, osteomyelitis, discospondylitis), and lymphadenitis (Davidson and Sykes 2021). However, a significant portion of infections are subclinical, complicating the detection and facilitating the spread of the zoonotic pathogen (De Massis et al. 2022; Kneipp, Deutscher et al. 2023). Diagnosis is challenging and relies on a combination of laboratory methods. While bacterial culture is the definitive gold standard, it might take up to 5 days and poses a significant biosafety risk. PCR in urine, blood, vaginal discharge, semen or fluids and tissue samples from aborted fetuses improves the detection speed, however, biosafety risk while processing these samples remains (WOAH Terrestrial Manual 2022). Sensitivity of these two pathogen detection methods is variable due to the pathogenesis of the disease and also depends on the protocols applied (Hollett 2006; WOAH Terrestrial Manual 2022). Therefore, serology plays an important role in the diagnosis of brucellosis (de Oliveira et al. 2011; Mol et al. 2020; Perletta et al. 2023). Serological tests for brucellosis are based on afore mentioned sLPS or rLPS, detecting antibodies against *B. abortus/melitensis/suis* and *B. canis/ovis* respectively. In dogs, as the more expected cause of brucellosis is *B. canis*, tests detecting antibodies against rLPS are utilized more commonly, including rapid slide agglutination test (RSAT), lateral flow test (LFT), indirect ELISA (iELISA). Agar gel immunodiffusion (AGID), complement fixation test (CFT) and immunofluorescence antibody test (IFAT) are used on a smaller scale (de Oliveira et al. 2011; Mol et al. 2020; Djokic et al. 2023; Perletta et al. 2023; LeCuyer et al. 2025).

In this study we present a canine brucellosis case caused by *B. suis* biovar 1 with a focus on diagnostic investigation.

## Case presentation

A 4-year-old, intact male, Cane Corso dog was referred to a veterinary clinic in Switzerland, due to loss of appetite, diarrhea in the previous days and intermittent vomiting in the previous weeks. Scrotal swelling had been observed by the owner for more than three months and a deslorelin implant did not lead to clinical improvement. Three other dogs of the same breed living in the same household for more than 6 months did not show any clinical signs. The dogs were fed a raw food diet using commercial raw food products from two different Europe-wide providers, as well as bones from a local butcher shop. The dog had been born in Germany but it has been living in Switzerland for at least the last two years prior to admission. The owners reported, that it had been to Italy two years before the admission but otherwise had not left the country.

In the clinical examination, fever (39.7 °C), monocytic leukocytosis (1.9 G/l monocytes (reference range: 0.6–1.12 G/l) of 17.2 G/l leukocytes (reference range: 5.05–16.76 G/l)), unilateral testicular and epididymal enlargement with tiger-stripe sign in ultrasonography, consistent with orchitis and epididymitis were detected. Following these findings, urine and blood samples were submitted to the Institute of Veterinary Bacteriology, Vetsuisse Faculty, University of Bern to assess canine brucellosis.

Real-time PCR (TaqMan™ Fast Advanced Master Mix, Applied Biosystems, Inc., Foster City, CA, USA) according to Hinic et al. for the targets *Brucella* spp. and *Brucella canis* (Hinić et al. 2008) from urine revealed a positive result for *Brucella* sp. (Ct value: 31) and late amplification for *B. canis* (Ct value: 38), which was initially considered positive as it was below the cut-off of 40 (Table 1).

**Table 1:** Summary of the samples and diagnostic test results

Sample	Method		Result
Serum	rLPS	LFT	negative
	sLPS	RBT^a^ iELISA^a^ CFT^a^	3+ S/P: 220% ≥ 1:128 (≥ 851 IU/ml)
Urine	real-time PCR^a^	*Brucella* spp.	positive (Ct: 31)
		*B. canis*	positive (Ct: 38)
	Culture followed by PCR^b^ and WGS from the isolate		*Brucella suis* biovar 1

Abbreviations: rLPS & sLPS, rough & smooth *Brucella* lipopolysaccharide-based test; LFT, lateral flow test; RBT, Rose Bengal Test); iELISA, indirect enzyme linked immunosorbent assay; CFT, complement fixation test; WGS, whole genome sequencing; S/P (sample/positive control).Notes: ^a^ 3 + refers to clear agglutination and positivity in RBT; S/*P* ≥ 120% is the threshold for positivity in iELISA; ≥20 IU/ml is the cut-off for positivity in CFT; Ct for positivity:40 in real-time PCR; ^b^ Bruce-ladder multiplex PCR (López-Goñi et al. 2011).

Serum tested negative in the rLPS based LFT (Bionote Antigen Rapid, C. Brucella Antibody Test Kit, Gyeonggi-do, Korea). However, serum reacted positive in sLPS based tests, namely Rose Bengal Test (RBT), iELISA (ID-Screen^®^ Brucellosis Serum Indirect Multi-species, BRU-MS, Montpelier, France) and CFT (Table 1). Commercial tests (LFT and BRU-MS iELISA) followed the instructions of the manufacturer and RBT and CFT were performed according to the manual of World Organization of Animal Health (WOAH Terrestrial Manual 2022) for farm animals and according to previous studies (Kneipp et al. 2023a, 2024), as there were no official guidelines for detection of antibodies against smooth *Brucella* spp in canine serum available at the time of writing. The utilized iELISA kit is intended for usage in farm animals. However, it is based on a protein binding to IgG universally, for which reason it can also detect canine IgG. Nevertheless, this kit has not been validated for canine serum to date, and we used the manufacturer’s threshold for farm animals.

Subsequently a culture of the sediment of 500 µl urine was performed under biosafety-level 3 conditions. The sample was streaked on trypticase soy agar II with 5% Sheep Blood (BD, Heidelberg, Germany) and brucella selective agar with previously published recipe (Abril et al. 2011) and incubated at 37 °C in 5% CO_2_ atmosphere using the BD GasPak EZ CO2 pouch system (BD, Heidelberg, Germany). More than 100 small uniform colonies were observed after 48 h on both agars. Since the serological tests indicated an infection with a smooth *Brucella* species and the initial real-time PCR result had been positive but with late amplification for *B. canis*, the isolate was further identified using the Bruce-ladder multiplex PCR assay according to López-Goñi et al., which allows to distinguish *B. suis* biovars and *B. canis* (López-Goñi et al. 2011). This analysis led us to identify the isolate (25Ref0017) as *B. suis* biovar 1 (Table 1) and shows that the initially applied Real-ime PCR cannot reliably distinguish *B. canis* from *B. suis*.

For whole genome sequencing (WGS), genomic DNA was isolated as described by Pitcher et al. (Pitcher et al. 1989) with slight modifications. The genomic DNA was then purified using the DNeasy PowerClean Cleanup Kit (Qiagen) and submitted to Microsynth AG (Balgach, Switzerland) for combined (Oxford Nanopore and Illumina) sequencing. This hybrid approach was chosen to achieve closed chromosome assemblies and ensure sufficient accuracy for SNP based analyses. Illumina sequencing was performed on a NovaSeq instrument with 250 bp paired-end reads. Oxford Nanopore sequencing achieved a mean read length of 3306 bp with an N50 of 7598 bp and a maximum of 55,567 bp. The mean read depth was 25x for nanopore and 330x for Illumina. Two complete circular chromosomes of 1,207,181 bp and 2,108,900 bp length were obtained and the sequence was submitted to GenBank (Accession No.: JBVMRO000000000).

**Fig. 1:**
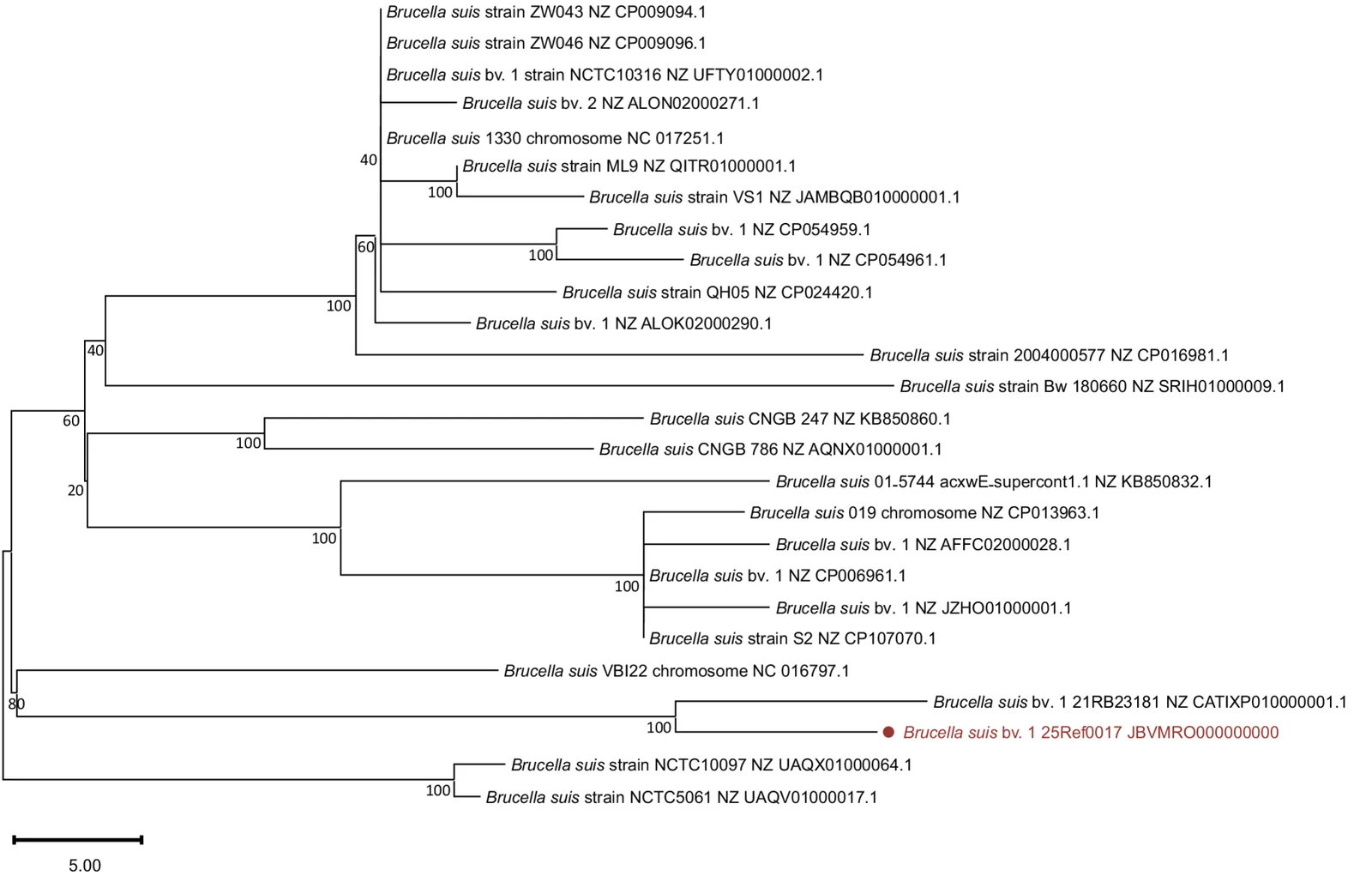
Phylogenetic tree based on core genome SNPs as determined by snippy v4.6.0 showing the relationship of the described strain, 25Ref0017 (marked red and with a dot) with the most closely related *Brucella suis* genomes available on GenBank. Evolutionary history was inferred using the Neighbor-Joining method. The optimal tree is shown. The percentage of replicate trees in which the associated taxa clustered together in the bootstrap test (10 replicates) are shown below the branches. The evolutionary distances were computed using the number of differences method and are in the units of the number of base differences per sequence. The rate variation among sites was modeled with a gamma distribution (shape parameter = 1). There was a total of 2,318,084 positions in the final dataset. Evolutionary analyses were conducted in MEGA11. The described isolate is most closely related to the dog isolate from Germany (21RB23181)

The sequence type was determined as ST-14 using MLST 2.0 (https://cge.food.dtu.dk/, accessed 30.01.2025). To compare the genome with *B. suis* sequences from GenBank a core genome single nucleotide polymorphism (SNP) based phylogenetic analysis was performed using snippy 4.6.0 and MEGA11 (Tamura et al. 2021) (Fig. 1). Isolate 25Ref0017 was found to be most closely related to strain 21RB23181 *B. suis* biovar 1, which was isolated in Germany, in 2021, also from a case of canine brucellosis (Aurich et al. 2023) (Fig. 1). However, the two isolates differ by 23 SNPs, two insertions, eight deletions and one complex variant, making them too distant for a direct epidemiological link.

Three other dogs living in the same household included a 4-year-old female, with whom the presented dog mated twice and produced one offspring from each mating; a 3-year-old male and a 6-month-old female. One serum of each of these three dogs was submitted in the following week after the diagnosis of the presented case and they all tested negative with RBT and BRU-MS iELISA. The owners were informed about the zoonotic risk but declined euthanasia of the affected dog, therefore treatment was attempted. Initially the dog had been treated with enrofloxacin (10 mg/kg, SID, *per os*) and carprofen (4 mg/kg, SID, *per* os). After five days following the brucellosis diagnosis, the treatment was changed to doxycyline (5 mg/kg, BID, *per* os, for 6 weeks) and rifampicin (10-15 mg/kg, SID, *per os*, for 4 weeks). The dog responded well to treatment and the scrotal swelling regressed drastically. A surgical castration following the antibiotic treatment was strongly recommended by the treating veterinarian, but this was declined by the owners. Furthermore, regular monitoring of urine and serological status of the affected dog as well as the three cohabiting dogs was deemed essential but was also declined by the owner.

### Discussion & conclusion

Canine brucellosis caused by *B. suis* biovar 1, has been reported frequently in Australia (Kneipp et al. 2023) and caused by biovar 1 or 2 sporadically in European countries such as Germany (Aurich et al. 2023), the Netherlands (van Dijk et al. 2018) and France (Girault et al. 2023). *B. suis* biovar 2 is known to circulate among wild boar and brown hare in Switzerland (Leuenberger et al. 2007; Abril et al. 2011; Wu et al. 2012) as in many European countries (Ruano et al. 2025). Therefore, detecting an infection with *B. suis* biovar 1 in a dog in Switzerland was unexpected for several reasons. First, *B. suis* biovar 1 has never been reported in wild animals in Switzerland and second, the presented dog is a companion animal without any association to hunting and without any history of travelling to a region, that is endemic for the agent. The only other locations, where the dog had been, were Germany and Italy, at least two years prior to admission to the veterinary clinic. Considering microscopic lesions usually appear five weeks post infection and the testicular enlargement in the following weeks (Davidson and Sykes 2021), it is unlikely that the dog acquired infection from these countries. Although we did not have the chance to investigate the commercial raw food products in this specific case, we know that they were purchased from two Europe-wide chain companies. Commercial raw food products had been either proven (van Dijk et al. 2018) or suspected (Aurich et al. 2023) as source of *B. suis* biovar 1 infection in dogs in Europe previously. While there are no genome sequences available for comparison from the case in the Netherlands presented by Dijk et al., both the isolate from a canine patient and the isolate from the component of the raw food product (the hare carcasses imported from Argentina by producer company), did show the same MLST sequence type as ours (ST-14). The isolate from the German case, 21RB23181 (Aurich et al. 2023), was the closest match with ours among the available genomes on GenBank even though they were too different for a direct epidemiological link. Although this observation is not proof, it does support the suspicion of commercial raw food products as source of infection in this case.

The higher zoonotic potential of *B. suis* biovar 1 naturally leads to the question of possible transmission to humans in contact with the affected dog, especially since the bacteria were shed in urine. Except for one caretaker from the clinical staff developing fever a few days after contacting the dog, which was confirmed to be COVID-19 infection, no one included in the clinical or diagnostic process developed signs consistent with brucellosis. The owner was recommended to have a medical check, especially serological testing, however, follow-up information was not available.

There is no laboratory protocol according to international animal health authorities for sLPS based serological methods for diagnosis of brucellosis in canine sera and only few validation studies for this purpose are available (Perletta et al. 2023; Kneipp et al. 2024). Additional to RBT and CFT, which are applied in these studies, we detected anti-brucella antibodies in our case by a commercial iELISA: BRU MS, which has previously not been used for canine serum. Complete validation of this kit for canine serum needs to be performed, however based on the clear positive result of this one serum as well as the negative results of the contact dogs which were confirmed by RBT, the threshold for positivity recommended by the manufacturer might also be suitable in canine serum.

Previous studies about *B. suis* infection in dogs (Kneipp, Deutscher, et al., 2023; Kneipp, Rose, et al., 2023) mention low detection rate from urine either by culture or PCR explaining the low level of horizontal transmission due to less successful excretion of this, rather swine adapted pathogen, among dogs. However, in our case we observed a high number of bacteria in urine. The reason for this observation remains unclear. It also remains unexplained why other dogs in the same household did not develop disease and were seronegative, despite the shedding of the bacterium in urine by the affected dog and despite the common food offered by the owner. However, it needs to be pointed out that the serology in all the dogs could be performed only once, and no follow-up data is available.

This case also points out a gap in the official legislations concerning zoonotic pathogens in companion animals. Brucellosis in farm animals is strictly controlled and surveyed in European countries. Despite increasing awareness and control measures, there are still gaps in the mandatory measures about canine brucellosis, even if it is caused by a highly zoonotic *Brucella* species. Transmission of *B. suis* as well as *B. canis* from dogs to humans is mostly associated with abortion of infected bitches, where high numbers of bacteria are shed (James et al. 2017; Kneipp et al. 2023a). However, there are human cases caused by *B. canis* of unclear exposure to non-breeding dogs and it is important to point that *B. canis* infection in human is underdiagnosed and under-investigated (Weese and Weese 2025). Considering the close contact between people with their dogs and the high ratio of subclinical carrier dogs, brucellosis in dogs constitutes a public health concern, which urges strict measures (Dadar et al. 2025; Djokic et al. 2023; Middlemiss 2021; Santos et al. 2021). Although the recommended approach in confirmed infected and especially sick animals is euthanasia, if this option is rejected, alternatively, long-term combined antibiotic therapy with or without neutering can manage these dogs clinically (James et al. 2017; Kneipp et al. 2023a). Such dogs should be routinely tested and have limited contact with other dogs and people as they might pose a risk as reservoir for the rest of their lives (Middlemiss 2021; Boyden 2022). This is especially relevant in the presented case of an infection with a non-endemic *B. suis* biovar, risking the spread of this more pathogenic biovar to domestic and wild animals in Switzerland. This contrasts with Australia where *B. suis* biovar 1 is endemic and treatment of affected dogs is considered a legitimate option (James et al. 2017).

Awareness about canine brucellosis is especially important among dog breeders, where the disease can cause dramatic results (Keid et al. 2017; Weese et al. 2020; De Massis et al. 2022), which underscores the relevance of routine screening of the breeding dog population. It is also highly important, that medical experts including veterinary and human physicians as well as laboratory diagnosticians be aware of this less expected pathogen with a high zoonotic potential.

## Data Availability

Whole genome sequence of the isolate is available in GenBank wit the accession number JBVMRO000000000.
